# Immigration modulates audiovisual emotional processing in adults: is this really an influence of the host culture?

**DOI:** 10.3389/fpsyg.2025.1533274

**Published:** 2025-01-29

**Authors:** Anna K. Nakamura, Hisako W. Yamamoto, Sachiko Takagi, Tetsuya Matsuda, Hiroyuki Okada, Chiaki Ishiguro, Akihiro Tanaka

**Affiliations:** ^1^School of Arts and Sciences, Tokyo Woman’s Christian University, Tokyo, Japan; ^2^Japan Society for the Promotion of Science, Tokyo, Japan; ^3^Department of Psychology, Ritsumeikan University, Ibaraki, Japan; ^4^Faculty of Human Science, Tokiwa University, Mito, Japan; ^5^Brain Science Institute, Tamagawa University, Machida, Japan; ^6^Faculty of Liberal Arts, University of the Sacred Heart, Tokyo, Japan

**Keywords:** immigration, audiovisual processing, emotion perception, functional MRI, right posterior superior temporal gyrus

## Abstract

**Introduction:**

Individuals from Western cultures rely on facial expressions during the audiovisual emotional processing of faces and voices. In contrast, those from East-Asian cultures rely more on voices. This study aimed to investigate whether immigrants adopt the tendency of the host culture or whether common features of migration produce a similar modification regardless of the destination.

**Methods:**

We examined how immigrants from Western countries to Japan perceive emotional expressions from faces and voices using MRI scanning.

**Results:**

Immigrants behaviorally exhibited a decrease in the influence of emotions in voices with a longer stay in Japan. Additionally, immigrants with a longer stay showed a higher response in the posterior superior temporal gyrus, a brain region associated with audiovisual emotional integration, when processing emotionally congruent faces and voices.

**Discussion:**

These modifications imply that immigrants from Western cultures tend to rely even less on voices, in contrast to the tendency of voice-dominance observed in native Japanese people. This change may be explained by the decreased focus on prosodic aspects of voices during second language acquisition. The current and further exploration will aid in the better adaptation of immigrants to a new cultural society.

## 1 Introduction

Emotion perception is a crucial aspect of social interaction. Although numerous studies have focused on emotion perception through a single cue (e.g., face, voice, or body), emotions are often expressed in a multimodal fashion in our daily lives. We perceive the emotional states of others by integrating multimodal cues, which are mostly congruent (e.g., a smiling face with a happy voice) but sometimes incongruent (e.g., a smiling face with an angry voice) to each other.

Multimodal emotional recognition, especially the integration of emotions from faces and voices, is known to be affected by cultures. Several studies have reported a cultural difference between East-Asian and Western cultures (Tanaka et al., 2010; Liu et al., 2015; Kawahara et al., 2021; Yamamoto et al., 2020). While East-Asian individuals tend to rely on vocal expressions when audiovisually integrating emotions, those from Western cultures tend to rely on facial expressions. Tanaka et al. (2010) examined how Japanese and Dutch participants recognize emotions when the emotions inferred from faces and voices were incongruent with each other. They found that Japanese participants had difficulty ignoring vocal expressions, whereas Dutch participants were more focused on facial expressions. It is also found that North Americans are more face-dominant than Chinese participants (Liu et al., 2015). In this paper, we define a tendency to be affected more by voices in East Asians as “voice-dominance” and the tendency to be affected more by faces in Westerners as “face-dominance,” based on relative differences.^1^

In this globalized world, numerous people migrate to different cultural societies upon adulthood. How does migration from one culture to another impact emotional perception? Two studies show that migrants from a voice-dominant culture (China) to a face-dominant culture (North America) acquire a face-dominant tendency, especially when they are highly exposed to the society in the new culture (Liu et al., 2017; Chen et al., 2022). A plausible explanation of this modification in immigrants is that immigrants from voice-dominant to face-dominant cultures acquire face-dominance in the process of adjusting to a face-dominant culture after immigration.

It has been supported in several research areas of perceptual/cognitive aspects that immigrants adopt tendencies common to the host culture. Regarding contextual processing, people in the United States are more accurate in ignoring the context when drawing a line in a square, while participants from Japan are more capable of incorporating contextual information (Kitayama et al., 2003). Their data from individuals engaged in a non-native culture (Japanese in the United States, Americans in Japan) showed similar cognitive tendencies to that is common in the host culture, not in their native culture. Athanasopoulos et al. (2010, 2011) examined the color perception of immigrants from cultures that distinguish lighter and darker shades of blue (e.g., Greece, Japan) to cultures that do not (e.g., UK). They found that immigrants become less sensitive to the differences in shades of blue with a longer stay or more frequent use of a language in no distinguishing culture, indicating that they are adopting the perceptual tendency in the host culture. Following these examples, immigrants may also show a similar tendency in the host culture in audiovisual emotional processing.

However, it is too early to fully support the idea that audiovisual emotional processing is modified as part of the adaptation process to the host culture. Previous studies only included immigrants from voice-dominant to face-dominant cultures to demonstrate the acquisition of face dominance (Liu et al., 2017; Chen et al., 2022) and no studies have been done on individuals experiencing migration in the opposite direction. If the immigrants from face-dominant to voice-dominant cultures show increased voice-dominance, this would support the possibility that changes in audiovisual emotional processing are a process of adaptation to the host culture. On the other hand, if the immigrants from face-dominant to voice-dominant cultures show rather increased face-dominance, a new possibility emerges that the immigrants become face-dominant during the emergence of a new culture regardless of the cognitive characteristics shared in the host culture. To examine these possibilities, this study aimed to elucidate how audiovisual emotion processing changes with the migration from Western face-dominant culture to East-Asian voice-dominant culture.

Although neuroimaging data can give us further insight into the modification of audiovisual emotion processing in immigrants, the study by Liu et al. (2017) is the only one that explored it so far. They examined the event-related brain potentials (ERP) associated with audiovisual emotional processing in immigrants from China to North America. These ERPs included N400, reflecting the process of identifying and evaluating emotional processes based on the speaker’s voice and facial expression, and the visual mismatch negativity (vMMN), reflecting the primary visual detection process. ERP data showed that the brain activities of immigrants were consistent with the patterns observed in resident Chinese participants, but not with resident North Americans. While the behavioral tendencies in audiovisual emotion processing were changing in the immigrant group, they could not find the relevant modification in brain activity.

To also explore the modification in the audiovisual emotion processing in the brain, this study examined the neural activity associated with the modification of audiovisual emotional processing during migration using functional magnetic resonance imaging (fMRI), which enables investigation of brain activities in broader brain regions with higher spatial resolution. Accumulating evidence indicates a role of the posterior superior temporal gyrus (pSTG) in audiovisual emotional processing, as well as roles of the thalamus, amygdala, and inferior frontal gyrus (Kreifelts et al., 2007; Park et al., 2010; Deen et al., 2020). Particularly, the right pSTG has been at the center of the discussion (Müller et al., 2011; Ethofer et al., 2013; Davies-Thompson et al., 2019), supported by a recent meta-analysis highlighting this area as the dominant region in audiovisual emotional integration (Gao et al., 2019). As the right pSTG has been proposed as the region primarily involved in audiovisual emotional integration, we hypothesized that activation in the right pSTG can also be modified with migration along with the behavioral responses.

Overall, this study explored the impact of immigration on audiovisual emotional processing and related neural responses using fMRI, focusing on immigrants from Western countries (face-dominant culture) to Japan (voice-dominant culture). The tendency of audiovisual emotion processing was measured by following the methods of Tanaka et al. (2010). To explore the change of behavioral and neural response in audiovisual emotion processing, correlations were analyzed between the duration of the stay in Japan (post-immigration period) and the tendency of face-dominance and voice-dominance in immigrants when processing Japanese faces and voices.

## 2 Materials and methods

### 2.1 Participants

Right-handed immigrants to Japan from Europe or North America were recruited via bulletin boards, mailing lists, social networking services, and snowball sampling. We could not conduct a power analysis based on previous related studies, as they mainly focused on a group comparison rather than the correlation between demographic characteristics and behavioral/neural data. Consistent with previous studies involving in group correlation analyses of neural data for the post-immigration period (Derntl et al., 2009; Liu et al., 2017), we recruited 29 immigrants. Five participants were excluded because of excessive head movements during fMRI scanning. One participant was excluded because of inadequate response in the audiovisual emotion task. Additionally, one participant with mixed Japanese and Western descent, possibly familiar with both cultures, was excluded. The outlier data from another participant were discarded, as their score on the Japanese vocabulary test based on lists from the Japanese-Language Proficiency Test (JLPT) (available at http://www.tanos.co.uk/jlpt/jlpt1/) was lower than the mean – 2 SD of the sample, indicating a low understanding of Japanese. Ultimately, data from 21 participants (5 women, 16 men; mean age ± SD: 25.14 ± 2.92 years, range: 21–30 years; 17 of which were university students) were included in the analysis.

The mean post-immigration period was 19.88 ± 15.87 months (range: 1.5–48 months). The participants emigrated from the United States (*N* = 5), France (*N* = 5), Canada (*N* = 2), Finland (*N* = 1), Sweden (*N* = 1), Germany (*N* = 1), Belgium (*N* = 1), Poland (*N* = 1), Hungary (*N* = 1), Russia (*N* = 1), Belarus (*N* = 1), and New Zealand (*N* = 1). The mean score of the JLPT Can-do Self-Evaluation List (available at https://www.jlpt.jp/e/about/candolist.html, the maximum score was 20) was 12.76 ± 6.13 (range: 1–20). All participants had normal or corrected-to-normal vision and no neurological disorders.

### 2.2 Audiovisual emotion task

#### 2.2.1 Stimuli

**Figure 1:**
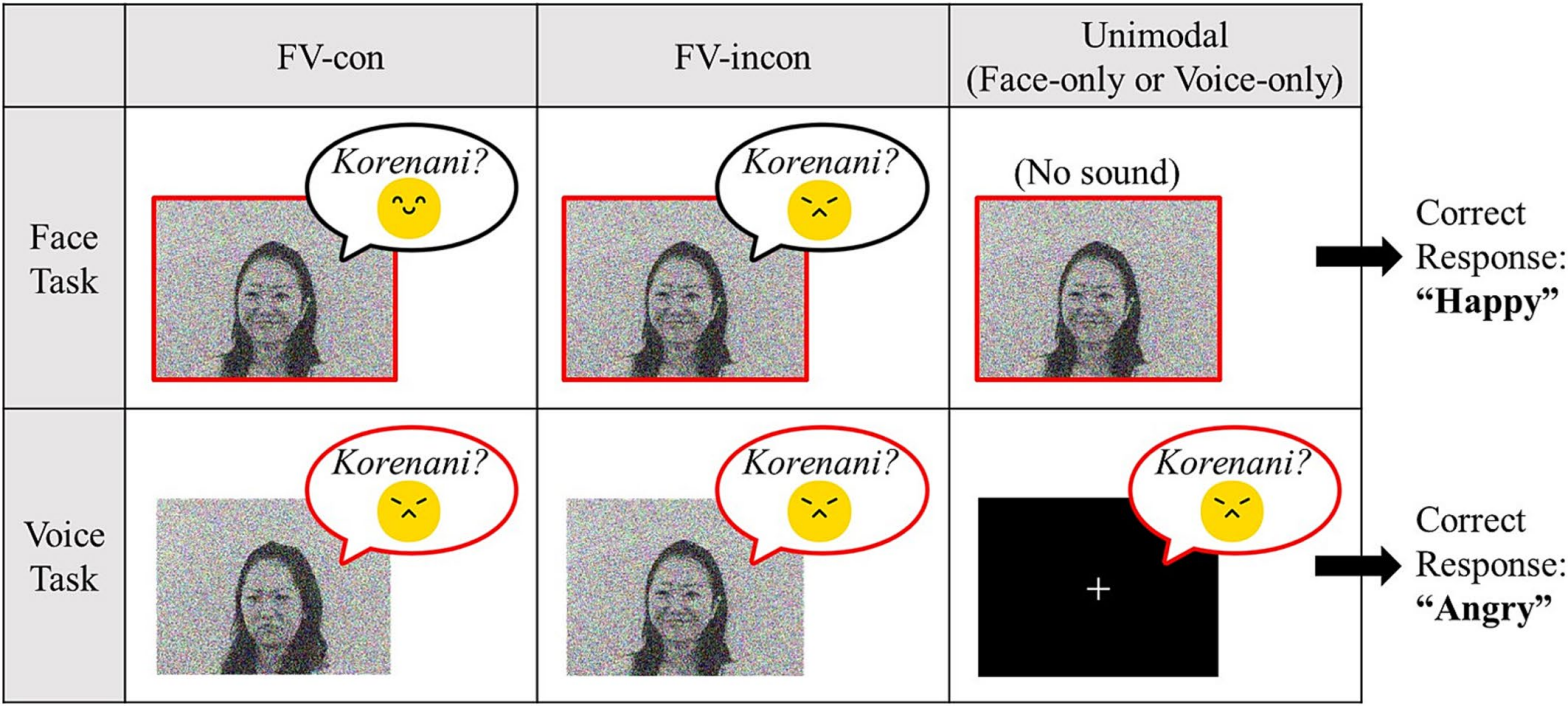
Description of task conditions and examples of Japanese stimuli. Participants were required to answer based on the emotion enclosed by the red bold lines in each task (faces in the face task, voices in the voice task).

The audiovisual stimuli were short videos originally used by Tanaka et al. (2010) (Figure 1). In these videos, Japanese and Dutch female models expressed happiness or anger through facial expressions and vocal utterances’ prosody. Two native-speaking models per language appeared in the videos. The linguistic information of each utterance was emotionally neutral: i.e., “Hello” (“*Hai, moshimoshi*” in Japanese, “*Hallo, dat ben ja*” in Dutch), “Goodbye” (“*Sayonara*” in Japanese, “*Een goede dag*” in Dutch), “What is this?” (“*Korenani*” in Japanese, “*Hey, wat is dit?*” in Dutch), and “Is that so?” (“*Sounandesuka*” in Japanese, “*Oh, is dat zo?*” in Dutch).

In addition to these audiovisually congruent stimuli, we created incongruent stimuli by dubbing the angry voices into the happy face videos, and the happy voices into the angry face videos. As for unimodal conditions, face-only stimuli were created by muting the sounds, while voice-only stimuli were created by substituting the video with a black background and a white fixation cross. Thus, there were four types of stimuli: face-voice congruent (FV-con), face-voice incongruent (FV-incon), face-only, and voice-only. In total, 128 types of stimuli were created: four types (FV-con, FV-incon, face-only, voice-only) × two languages (Japanese and Dutch) × two emotions (angry and happy) × two models × four utterances.

In the preliminary experiment by Tanaka et al. (2010), the rate at which facial expressions were correctly identified (i.e., face-only stimuli) was higher than the rate observed for voices (i.e., voice-only stimuli) using the original video clips. Therefore, we decreased the visibility of the videos to equate the difficulties of emotional judgment between faces and voices by adding salt-and-pepper noise at a density of 0.72 using MATLAB 2018a (MathWorks, Natick, MA, United States). We adopted this noise density level because our preliminary experiment with Japanese native speakers (undergraduate and graduate students) indicated that the average correct judgment rates for face-only stimuli with 0.72 density level noise did not significantly differ from that for voice-only stimuli [face-only stimuli: 82.4%, voice-only stimuli: 80.6%, *t*(18) = 0.54, *p* = 0.60].

#### 2.2.2 Task procedure

The audiovisual emotion task was conducted during MRI scans, controlled using Presentation^®^ software (Neurobehavioral Systems Inc., San Francisco, CA, United States). The audiovisual stimuli were presented using an EIZO monitor (EV2450) and N.K.Y. headphones.

**Figure 2:**
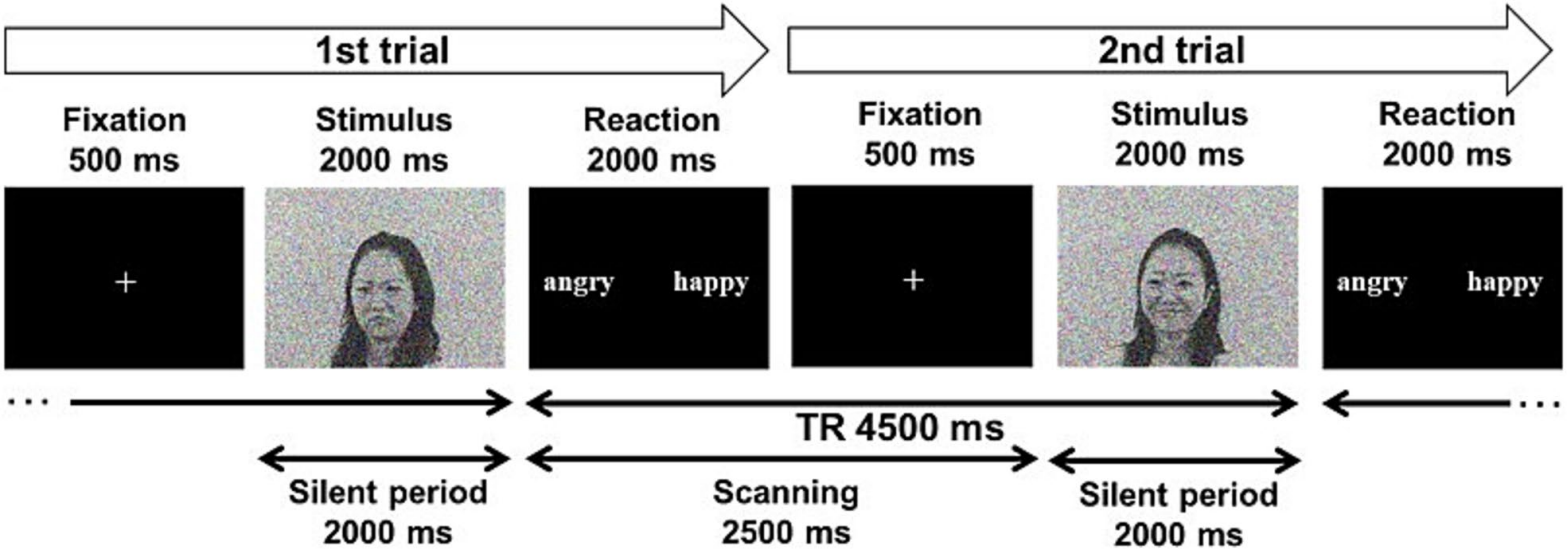
Task and scan procedure.

The audiovisual emotion task consisted of two task conditions: the voice and face tasks. In the face task, participants were required to estimate the model’s emotion based on her face while ignoring the emotional content of the voice. In the voice task, participants were asked to estimate the model’s emotion based on their voice while ignoring the emotion in the face. Each task condition included congruent, incongruent, and unimodal conditions (face-only in the face task, voice-only in the voice task). An audiovisual or unimodal stimulus was presented for 2 s in each trial, followed by a 2-s response phase. Response alternatives (“angry” and “happy”) were presented on the screen, and the participants pressed the corresponding key to respond (Figure 2). A 500-ms fixation cross followed the response phase, and the video proceeded to the subsequent trial. The participants completed two blocks for each task condition; the task order was Face-Face-Voice-Voice or Voice-Voice-Face-Face. In each block, 32 congruent, 32 incongruent, and 32 unimodal stimuli (face-only in the face task, voice-only in the voice task) were presented, including Japanese and Dutch models. The face-only stimuli were not included in the voice task, and vice versa. Additionally, we included 16 null trials per block in which the participants were presented with a fixation cross instead of a video clip for 4 s for baseline measurement. The participants were not required to respond to null trials. All trials were presented in a pseudo-random order. Therefore, the face and voice tasks included 224 trials each [(96 trials +16 null trials) × 2 blocks], for 448 trials in total.

#### 2.2.3 Converting variables

As the goal of this study was to examine the effect of host culture on the audiovisual emotion processing in immigrants to Japan, we only focused on the conditions with Japanese stimuli in the analysis. The accuracy rate was computed for each stimulus type for each task condition for each participant. Then, the difference in accuracy between multimodal (FV-con, FV-incon) and unimodal conditions (face-only for the face task, voice-only for the voice task) was calculated to understand the tendency in audiovisual emotion processing. Differences in the accuracy between multimodal and unimodal conditions will enable us to differentiate the effect of the emotional congruency of the presented voice when face processing in the face task, and vice versa in the voice task. The increased accuracy in the FV-con conditions compared to the unimodal conditions reflects the facilitation effect of the irrelevant modality in each task. If the accuracy of the FV-con condition in the face task minus that of the face-only condition was positive, the difference in these accuracies reflects the degree of facilitation effect of an emotionally congruent voice on emotional perception from the face. Likewise, if the accuracy of the FV-con in the voice task exceeded the accuracy of the voice-only condition, the difference in these accuracies can be regarded as a facilitation effect on voice recognition by a congruent face. On the other hand, the decreased accuracy in the FV-incon conditions compared to the unimodal conditions reflects the inhibition effect of the irrelevant modality in each task. If the accuracy of the FV-incon in the face task was less than the accuracy in the face-only conditions, the difference in these accuracies reflects an inhibition effect on face recognition by an incongruent voice. If the accuracy of FV-incon in the voice task was lower than the accuracy in the voice-only condition, the difference in these accuracies can be regarded as an inhibition effect on voice recognition by an incongruent face.

### 2.3 fMRI scanning

#### 2.3.1 fMRI image acquisition

A 3-Tesla MR system (Trio, Siemens, Erlangen, Germany) was used to acquire T2*-weighted gradient echo-planar images (EPI) with blood oxygenation level-dependent (BOLD) contrast. Forty-two EPI slices were acquired for each volume (voxel size: 3.0 × 3.0 × 3.5 mm^3^; repetition time: 4,500 ms; echo time: 30 ms; flip angle: 80°; field of view: 192 mm^2^). The sparse temporal sampling method (Hall et al., 1999) was adopted to reduce the effect of the scanner’s noise as the task included auditory stimuli (Figure 2). We discarded the first three volumes to compensate for the counterbalanced T1 saturation effects. A high-resolution anatomical T1-weighted image (MPRAGE; voxel size: 0.75 × 0.75 × 1.0 mm^3^; repetition time: 2,500 ms, echo time: 4.38 ms; flip angle: 8°; 256 × 256 matrix; 192 slices) was also acquired for each participant.

#### 2.3.2 Data processing

The fMRI data were preprocessed and analyzed using SPM12 (Welcome Centre for Human Neuroimaging, London, UK) on MATLAB R2020a. The SPM12 toolbox MarsBaR (0.44) was used to create ROI masks.

The fMRI data were subjected to the following preprocessing methods: head movement corrections, slice time adjustments, coregistration to T1-weighted image, spatial normalization to the standard Montreal Neurological Institute (MNI) template, and smoothing. After preprocessing, a two-level approach was adopted in SPM12. Regressors were added for two languages by three conditions (congruent, incongruent, unimodal), adding null conditions. Each regressor was generated by adopting a canonical hemodynamic response function with a two-second duration. The onset of keypress, visual stimuli other than faces (response choices), and stimuli with missed responses were also included in the model as variables of no interest. Head movements computed through the realignment process were also included for motion correction. For the group-level analysis, contrasts of [FV-con – unimodal] and [FV-incon – unimodal] were created for each task condition with Japanese models.

Since the neural activity in the right pSTG is supposedly related to audiovisual emotional processing (Gao et al., 2019), we performed a region-of-interest (ROI) analysis using two right pSTG masks as described in a recent meta-analysis (Gao et al., 2019). Two right pSTG ROI masks were created as 8-mm spheres (peak MNI coordinates of ROI 1: 52, −44, 8; ROI 2: 62, −50, 6).

We then conducted a whole-brain analysis to explore whether each contrast estimate of [FV-con – unimodal] and [FV-incon – unimodal] in each task (face task and voice task) in any brain region correlated with the duration of the post-immigrant period.

### 2.4 Statistical processing and analysis

The correlations were calculated between the behavioral responses and the post-immigration period to examine how the tendency of audiovisual emotional processing changes with the duration of stay in Japan. If migrants adopt the audiovisual emotional processing tendency of the host cultural environment, the post-immigration period should be characterized by increased voice-dominance. In contrast, if immigrants become face-dominant regardless of the host culture, the period should be characterized by increased face-dominance. Nonparametric Spearman’s correlation coefficient, rs, was adopted for all correlation analyses because the Shapiro–Wilk test showed that the post-immigration period did not follow normal distributions (*p* = 0.029).

The fMRI data from ROI analysis were also subject to correlation analysis with the post-immigration period to see the modulation in the right pSTG related to the change during the stay. The following whole brain analysis was aimed to explore other brain regions that relate to the change with a longer stay. The statistical threshold was set uncorrected *p* < 0.001 at the voxel level and *p* < 0.05, family-wise error (FWE) corrected at the cluster level, assuming the whole brain as the search volume.

## 3 Results

### 3.1 Behavioral data

**Figure 3:**
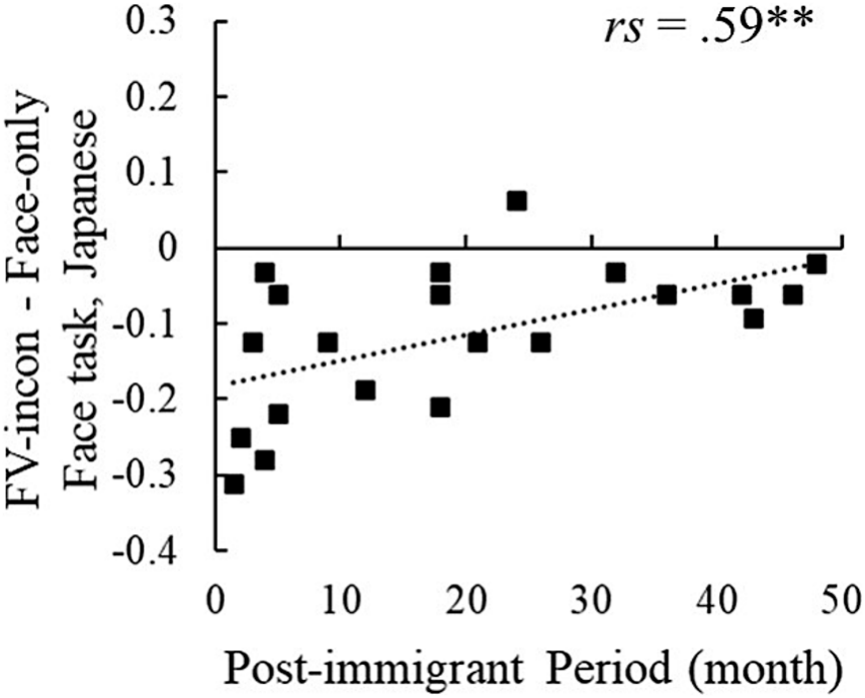
The post-immigration period and the difference in accuracy rate of FV-incon and Face-only in the face task. **p < 0.01.

The correlation analysis revealed that the difference in accuracy between FV-incon and face-only conditions in the face task was significantly correlated with the post-immigrant period (*rs* = 0.59, *p* = 0.005), while the difference between FV-con and face-only conditions was not (Table 1; Figure 3) (see Supplementary Figure S1 for the accuracy data in each condition). No statistically significant correlations were found between the post-immigrant period and the responses in the voice task. The difference between FV-incon and face-only conditions in the face task reflects the inhibition effect of emotionally incongruent voices on facial emotion processing. Its increase with a longer stay indicates that immigrants from Western to East-Asian cultures become less susceptive to emotionally incongruent voices when processing emotions on Japanese faces.

**Table 1:** Descriptive statistics and correlations for the difference in accuracy (0 ~ 1) between multimodal and unimodal conditions.

		Descriptives		Correlation (rs)
Task	Variable	Mean	SD	Post-immigration period
Face	FV-con – Face-only	0.01	0.06	−0.02
	FV-incon – Face-only	−0.12	0.10	0.59***
Voice	FV-con – Voice-only	0.08	0.06	0.25
	FV-incon – Voice-only	−0.10	0.13	0.30

***p < 0.005.

### 3.2 fMRI data: right pSTG ROI analysis

**Figure 4:**
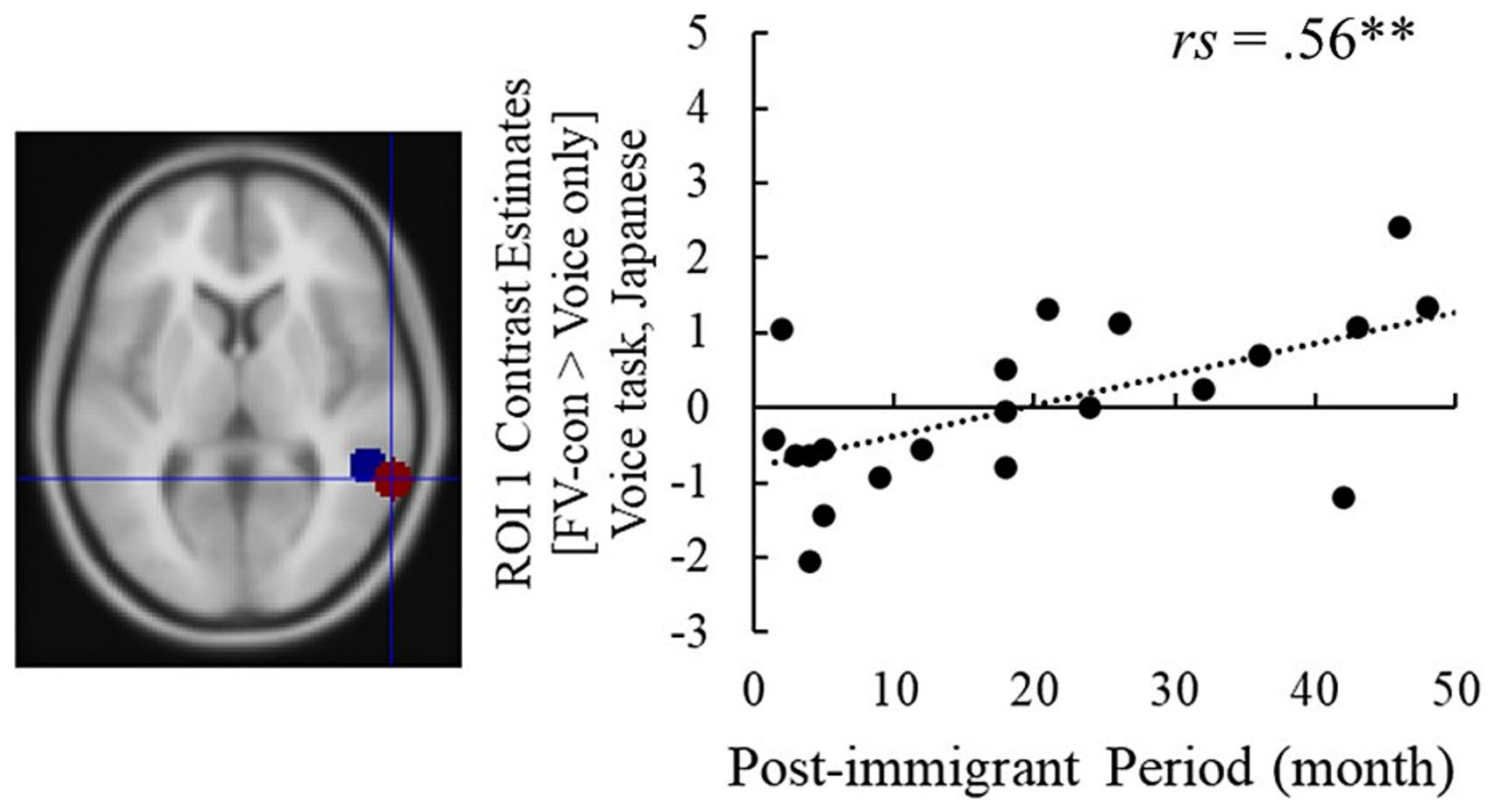
The post-immigration period and the right pSTG activity in ROI 1 in the voice task [FV-con – Voice only]. **p < 0.01. The red dot in the brain image is ROI 1 and the blue one is ROI 2.

Contrast estimates of [FV-con – face-only] and [FV-incon – face-only] in the right pSTG ROI underwent correlation analysis with the post-immigration period (Table 2) (see Supplementary Figure S2 for basic validation of the fMRI data). Regarding the face task, no statistically significant correlations with the post-immigration period were detected for both contrasts. On the other hand, the contrast estimate of [FV-con – voice-only] in the voice task was significantly positively correlated with the post-immigrant period (ROI 1: *rs* = 0.56, *p* = 0.009; ROI 2: *rs* = 0.53, *p* = 0.013), while [FV-incon – voice-only] showed no statistically significant correlations (Figure 4). These results indicate that the pSTG activity when processing voices presented with emotionally congruent faces increases with the length of stay.

**Table 2:** Descriptive statistics and correlations for each contrast estimate (beta value).

		Right pSTS ROI	Descriptives		Correlation (rs)
Task	Contrast		Mean	SD	Post-immigration period
Face	FV-con – Face-only	1	1.02	1.06	0.15
		2	0.65	1.52	−0.18
	FV-incon – Face-only	1	1.05	1.21	0.20
		2	0.74	1.94	−0.01
Voice	FV-con – Voice-only	1	0.02	1.11	0.56**
		2	0.04	1.53	0.53*
	FV-incon – Voice-only	1	0.27	0.88	0.25
		2	0.28	1.16	0.15

**p < 0.01, *p < 0.05.

### 3.3 fMRI data: whole-brain analysis

We conducted a whole-brain analysis to broadly detect the brain region related to the modification in audiovisual emotion processing observed following immigration. Including both contrast of [FV-con – unimodal] and [FV-indon – unimodal] in both face and voice tasks, no regions where the activity of audiovisual emotion processing significantly correlated with post-immigrant duration. These results indicate that no change in activity in any region during audiovisual emotion processing was detected that varied with the length of the stay. While the activity in the right pSTG was correlated with the post-immigrant period in ROI analysis, the whole-brain analysis showed otherwise. The results showed that the relation with the change of activity in pSTG during the migration is not strong enough to be replicated in the whole-brain analysis, which is exploratory and has a stricter threshold of detection.

## 4 Discussion

The present study aimed to examine audiovisual emotional processing in immigrants from Western countries to Japan by applying an fMRI task. Behaviorally, immigrants were less distracted by emotionally incongruent voices during face processing compared with immigrants with a short-term stay. This finding indicates that for immigrants from a Western face-dominant culture to an East-Asian voice-dominant culture, the voice component becomes even less relevant as the duration of stay increases.

Our behavioral data was consistent with the previous studies in the point that the audiovisual emotion processing does change with migration, while the direction of the change was not consistent with that expected from their discussions (Liu et al., 2017; Chen et al., 2022). Both studies focused on the immigrants from East-Asia voice-dominant culture to Western face-dominant culture to find out the increased face-dominance. They discussed the possibility that immigrants adopt the emotion-processing style in the host culture in the same way that other perspective/cognitive aspects of the immigrants also assimilate the tendency of the host culture (Athanasopoulos et al., 2011; Athanasopoulos et al., 2010; Kitayama et al., 2003).

If the audiovisual emotion processing in immigrants is modified to assimilate with that in the host culture, the immigrants to East-Asian voice-dominant culture from Western face-dominant culture would experience an increase in voice-dominance. However, our data showed otherwise. The current data showed that immigrants from Western culture to Japan become less susceptible to the emotion in the voice, which was in contrast to the expected adoption of the voice-dominant tendency shared in Japan.

From the fMRI data, the activity of right pSTG when processing the voices presented with audiovisually congruent faces was increased in the immigrants with a longer stay. This result can be inferred that the immigrants become to integrate congruent faces more during voice processing, given that the right pSTG is the main region of audiovisual emotion processing (Müller et al., 2011; Ethofer et al., 2013; Davies-Thompson et al., 2019). Although the relationship in the right pSTG was not strong enough to be detected in the whole-brain analysis, no other region was detected to have a strong relationship with modifications in audiovisual emotion processing either.

In conjunction with our behavioral data, immigrants may come to depend on the emotional information from congruent faces when becoming insensitive to vocal prosodies. As a result, immigrants may begin to integrate faces with voices more during audiovisual emotion processing with a longer stay.

Current results suggest a possibility that immigration generally decreases the impact of voice-dominance regardless of the host culture. One possible explanation of the face-dominance in immigrants is the process of second language (L2) acquisition. Migration to a new cultural society requires people to learn new languages. Linguistic aspects of voices are almost always included in emotional expression from voices. Interestingly, developmental studies have demonstrated a trade-off between the comprehension of the linguistic content of speech and the perception of emotional prosody during native language acquisition. Prelinguistic infants exhibit a higher sensitivity to emotional prosody even in foreign language speech (Fernald, 1993), whereas 2- and 3-year-old children, whose language proficiency is still developing, are less attentive to the emotional prosodic cues of speech (Quam and Swingley, 2012). Moreover, even preschoolers and young school-aged children prioritize linguistic content over prosodic information until approximately age 10 (Friend, 2000). Thus, the impact of immersion in a new culture on the perception of the paralinguistic aspect of speech, specifically vocal expressions, should be considered. The research on audiovisual speech perception with bilingual speakers has also shown such a general effect of language or culture, revealing that experiences of non-native language led listeners to focus on faces (Marian et al., 2018). Individuals possibly tend to focus more on the linguistic aspect during L2 learning, potentially leading to a decrease in voice-dominant tendency and a relative increase in face-dominant tendency. Future studies are expected to explore this possibility further.

Although our behavioral data showed that the immigrants with longer stays are less affected by task-irrelevant voices when processing emotional faces (the face task), we could not detect the increased effect of task-irrelevant faces when processing voices (the voice task). It is possible that the behavioral response in the current task was also affected by the change in inhibitory function in general. Accumulated evidence shows that bilingual people have higher inhibitory function (e.g., Bialystok and Craik, 2022). If immigrants with a longer stay show a higher ability to inhibit task-irrelevant information, the participants will be less affected by incongruent information in both face and voice tasks. If the inhibitory function was enhanced while the sensitivity to prosody expressions decreased, the congruency effect in the voice task is subjected to a negative change affected by inhibitory function and a positive change by the insensitivity to vocal expressions. That is, the ability to inhibit task-irrelevant facial expressions may offset the decrease in sensitivity to vocal prosody, resulting in a non-significant correlation. Although we did not measure each participant’s inhibition skills, considering previous studies focusing on a relationship between bilingualism and inhibitory function, future research investigating the link between inhibitory function and these findings is warranted.

Despite our findings, there are several limitations in this study. First, the post-immigration period of the participants in this study was relatively short compared to that in previous studies (Liu et al., 2017; Chen et al., 2022), which involved periods of 6–12 years and 9–26 years, respectively. We could not determine how long the decrease in voice-dominance lasts concerning the L2 learning period and intergroup experiences, and whether or when immigrants start to adopt the style of audiovisual emotional processing of the host culture remains unknown. We speculate that the effects of the changes generally associated with immigration are relatively short and that culture-specific effects prevail after that. In detail, the voice-dominance of immigrants from Western to East-Asian cultures may be characterized by an initial decrease because of interference in the processing of emotional prosody during L2 learning, followed by an increase as they become sufficiently competent to process both the linguistic and prosodic aspects of the voice, reflected as a U-shaped curve. Face-dominance exhibited no significant correlation with the duration of the post-immigration period in our study, possibly because of the balancing effect from immigration itself (i.e., increased exposure to Japanese facial expressions and the culture-specific effect of inhibiting the less reliable information from Japanese faces). Immigrants to Japan may develop an even lower face-dominance if the culture-specific effect begins to exert a greater influence on audiovisual emotional processing as the length of stay increases. Further exploration is warranted to reveal how audiovisual emotional processing changes at the behavioral level in the long term, as well as the corresponding neural modifications.

Secondly, to differentiate the facilitation and inhibition effect in audiovisual emotion processing in the brain, the current study focused on the comparison between multimodal and unimodal responses but not on the comparison between congruent and incongruent conditions. The comparison of responses between congruent and incongruent (Jeong et al., 2011; Müller et al., 2011) and multimodal and unimodal (Pourtois et al., 2005; Kreifelts et al., 2010; Kokinous et al., 2015) are both widely examined in the neurological studies of audiovisual integration. One major advantage of the comparison of multimodal and unimodal responses is that the multimodal and unimodal comparisons make it possible to understand the facilitation effect of congruent stimuli and the inhibition effect of incongruent stimuli as two independent effects, not as the bipolar of one effect. This gives a clearer view of the change in audiovisual emotion processing, especially when comparing the audiovisual emotion processing between subjects with different lengths of stay after immigration. Although the multimodal and unimodal comparisons were the better option given the design and the aim of this study, care should be taken when directly comparing the results with those of other studies on audiovisual emotion processing.

Furthermore, the lack of a control group limits the validity of this study. Ideally, the immigrants’ responses should be compared with those of non-immigrants in the host and native cultures, as in the study by Liu et al. (2017), to provide a clear view of the characteristics of audiovisual emotional processing in immigrated persons. Although our data were underpowered to indicate the general effect of immigration on audiovisual emotional processing via a group comparison, we detected some changes by exploring the individual differences correlated with the duration of stay and language competency. We plan to obtain a more detailed understanding by integrating data from between-group and within-group comparisons. Lastly, we have to be cautious about the external validation of this study. There are considerable differences in emotional communications among East-Asian countries (Camras et al., 2006), and this study only represents the migration to Japan. It will be another research question to compare to which countries of East Asia they migrate.

## 5 Conclusion

The current study revealed that emotion in voices becomes even less relevant in audiovisual emotional processing in immigrants from a Western face-dominant culture to a Japanese voice-dominant culture. The fMRI data support this observation, as the modification in the right pSTG activity suggested that immigrants begin to integrate emotionally congruent faces during the processing of emotional prosody. This modification in audiovisual emotional processing may be explained more by the effect of immigration itself than the cultural influence from the host culture: When learning a new language, the linguistic aspect of the voice may interfere with the processing of emotional prosody. This study and subsequent analyses may contribute to understanding how immigrants communicate and adjust to a new culture, an increasingly relevant factor in this internationalized society.

## Data Availability

The raw data supporting the conclusions of this article will be made available by the authors, without undue reservation.

## Footnotes

^1^In addition to audiovisual emotion perception, cross-cultural studies have revealed a variety of differences in the integration of multiple cues. One of these is the cultural difference in the focus of attention: focal versus contextual information. East Asians tend to focus on background objects and contextual information, while Westerners are more likely to pay attention to focal objects in a scene (e.g., Chua et al., 2005; Imada et al., 2013; Masuda et al., 2008; Masuda and Nisbett, 2001). This difference has also been demonstrated in tasks involving voices (Ishii et al., 2003). Considering these studies, Westerners may focus more on faces as focal information, while Asians focus more on voices as contextual information. It is possible that cultural differences in audiovisual emotion perception would stem from such cultural differences in attention to focal versus contextual information. Nevertheless, here, we discuss the effect of culture on audiovisual emotion perception focusing on faces versus voices, as discussed by Tanaka et al. (2010), rather than focusing on focal versus contextual information for the following reason. In their paradigm, participants were asked to direct attention to a face or voice, making the other stimuli contextual information (e.g., in a task wherein participants were asked to identify a speaker’s emotion focusing on their voices, facial information was regarded as contextual information). Considering Japanese participants’ difficulty in ignoring voices and Dutch participants’ difficulty in ignoring faces regardless of the focus of attention, cultural differences in the focal versus contextual information alone cannot explain these results. Therefore, we regard the cultural difference in audiovisual emotion perception as the difference between face- and voice-dominance in this study, instead of interpreting it as the difference in the tendency of focusing on contextual/focal information.
