# Supplementary material for: Immigration modulates audiovisual emotional processing in adults: is this really an influence of the host culture?
Source: Front Psychol. 2025 Jan 29;16:1533274. doi: 10.3389/fpsyg.2025.1533274 (PMC11813912; doi:10.3389/fpsyg.2025.1533274)
Supplement: Supplementary file 1 [file Presentation_1.PDF]

Supplementary Material

1 Supplementary Figures

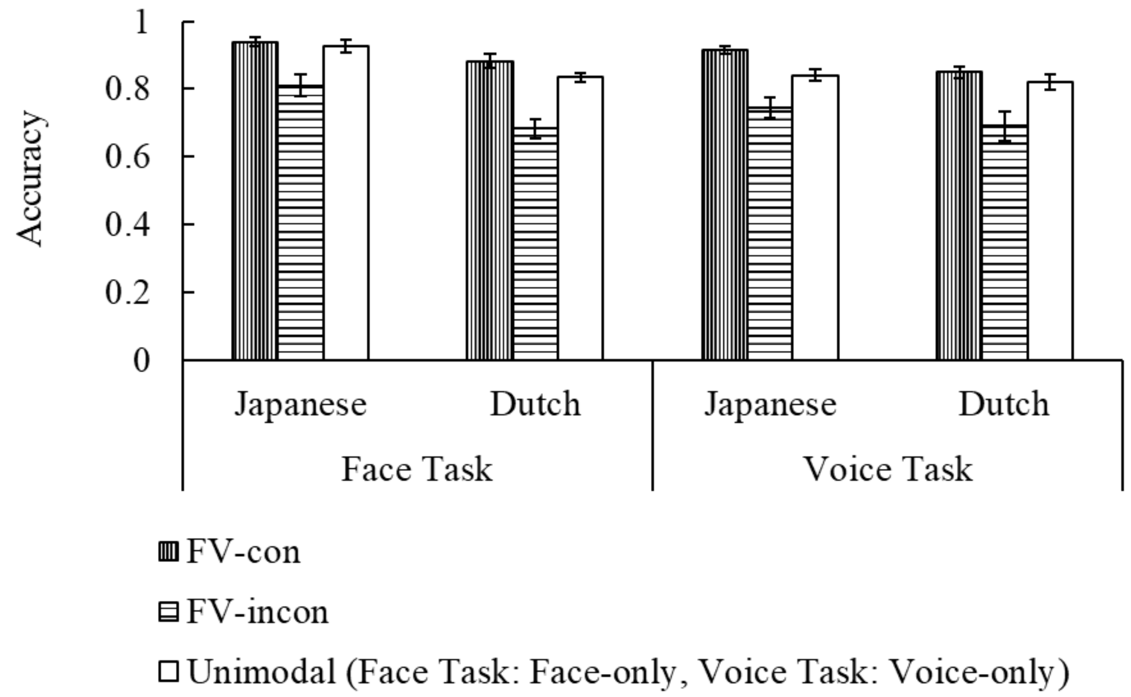

Supplementary Figure 1. Accuracy of behavioral responses in each condition. Error bars represent standard errors.

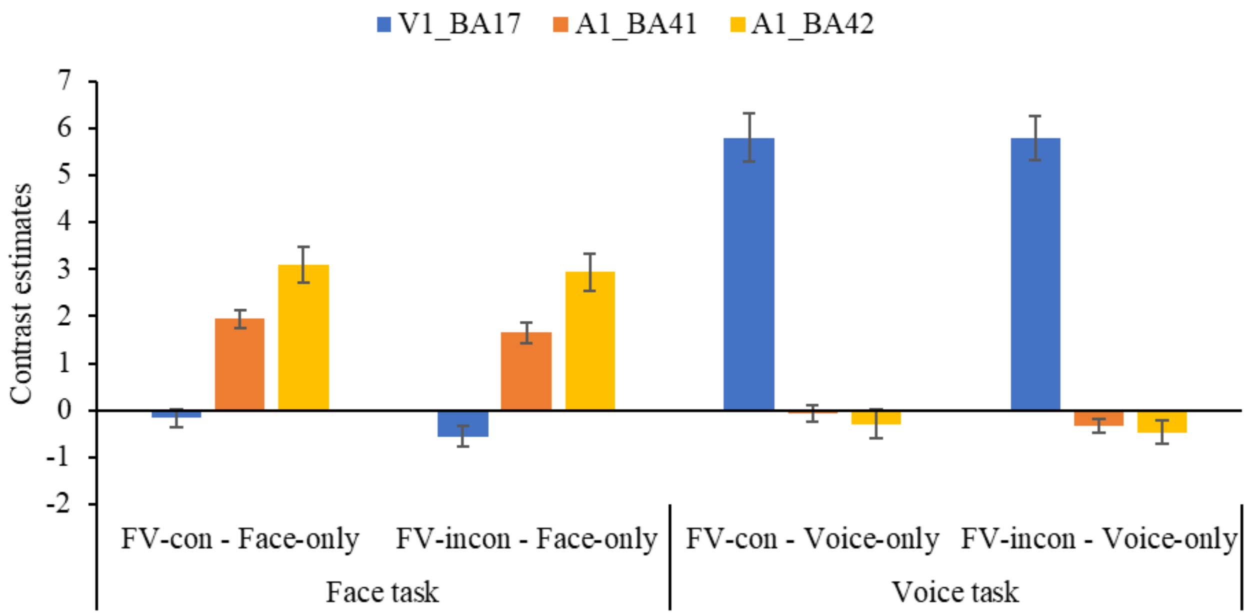

Supplementary Figure 2. Preliminary check of fMRI measurement. To check the basic validation of the fMRI data in this study, we conducted a ROI analysis by extracting BOLD signals from pre-defined ROIs. Contrasts of [FV-con - unimodal] and [FV-incon - unimodal] of both face and voice tasks were included in the analysis. As ROIs, bilateral Brodmann area (BA) 17 located in the primary visual cortex (V1), and bilateral BA 41 and BA 42, both located in the primary auditory cortex (A1), were included in the analysis. Masks for ROI analysis were created based on WFU_PickAtlas (https://www.nitrc.org/projects/wfu_pickatlas/) using MarsBaR version .44.
